# Micro RNA-155 plays a critical role in the initiation of food allergen-related inflammation in the intestine

**DOI:** 10.18632/oncotarget.18723

**Published:** 2017-06-28

**Authors:** Ri-Tian Lin, Jiang-Qi Liu, Hui-Ying Lu, Ya-Mei Chen, Li Guan, Zhi-Gang Liu, Zhan-Ju Liu, Ping-Chang Yang

**Affiliations:** ^1^ Department of Gastroenterology, The Shanghai Tenth People’s Hospital of Tongji University, Shanghai 200072, China; ^2^ The Research Center of Allergy & Immunology, Shenzhen University School of Medicine, Shenzhen 518060, China; ^3^ Department of Physical Examination, Affiliated Luohu Hospital, Shenzhen University, Shenzhen 518001, China

**Keywords:** food allergy, regulatory B cells, intestine, micro RNA, interleukin 13

## Abstract

The pathogenesis of food allergy (FA) is to be further investigated. Regulatory B cells (B10 cell) play a critical in the maintenance of the homeostasis in the intestine. Deregulation of B10 cell is associated with immune inflammation. Micro RNA (miR) 155 is involved in affecting immune cell function. This study tests a hypothesis that miR-155 affects the B10 cell function to facilitate the initiation of FA. In this study, BALB/c mice were sensitized to ovalbumin (OVA) to induce FA-like inflammation in the intestine. B cells were isolated from the intestine by magnetic cell sorting. The expression of miR-155 and IL-10 in B cells was assessed by real time RT-PCR. The results showed that mice sensitized to OVA showed FA-like inflammation and lower frequency of B10 cell in the intestine. B cells isolated from the intestine of FA mice showed higher levels of miR-155 and lower levels of IL-10. Although all the three T helper (Th)2 cytokines, including interleukin (IL)-4, IL-5 and IL-13, were higher in the serum, only IL-13 was positively correlated with the levels of miR-155 in the intestinal B cells. Exposure to IL-13 in the culture markedly increased the expression of miR-155 and suppressed the expression of IL-10 in B cells. Blocking miR-155 abolished the IL-13-induced IL-10 suppression in B cells and inhibited FA response in mice. In conclusion, miR-155 plays a critical role in the initiation of FA in mice. Blocking miR-155 has therapeutic potential in the treatment of FA.

## INTRODUCTION

It is estimated that the prevalence of food allergy (FA) is about 2-4% in adults and 4-6% in children [1]. Current therapies for FA are not satisfactory. Most FA cases rely on avoiding the ingestion of the offending foods [2]. Although FA generally does not cause serious injury in the body, it may induce life-threatening anaphylactic shock in some cases [3]. FA is also associated with the pathogenesis of some serious diseases, such as inflammatory bowel disease [4], chronic nephritis [5], etc. Thus, to elucidate the pathogenesis of FA is of significance.

The pathological feature of FA mainly includes a condition of T helper-2 (Th2) polarization in the body, including over production of specific IgE and Th2 cytokines, profound infiltration of mast cell/eosinophil in the intestine [6]. The specific IgE bind to the high affinity IgE receptor on the surface of mast cells to make mast cell sensitized. Upon re-exposure to specific antigens, the sensitized mast cells are activated to release preformed chemical mediators to initiate allergic symptoms [6]. Yes, how the skewed Th2 response initiated is not fully understood.

The immune regulatory cells, such as regulatory T cells (Treg) and interleukin (IL)-10-producing regulatory B cells (B10 cell), regulate the immune response in the body [7, 8]. Upon activation, Tregs and B10 cells release immune regulatory molecules, such as transforming growth factor (TGF)-β and IL-10, to suppress other immune cell activities. Recent research records have emphasized the role of B10 cells in suppressing CD4^+^ T cell activities [8]. B10 cells play a critical role in the maintenance of the homeostasis in the intestine [9, 10]. The Th2 polarization in the intestine implicates a condition of B10 cell. A decrease of B10 cell was found in FA patients [11]; the underlying mechanism to be further investigated.

Micro RNA (miR) is a single non-coding RNA chain with 18-22 nucleotides in length. Cumulative reports indicate that miRs are involved in the regulation of immune responses [12]. It is suggested that miR-155 can post-transcriptionally regulate the expression of IL-10 [13]. Whether miR-155 suppresses IL-10 in B cells in the initiation of FA remains to be further investigated. Thus, we hypothesize that miR-155 suppresses IL-10 in intestinal B cells that plays an important role in the initiation of FA. In this study, we observed the role of miR-155 in the development of FA in a mouse model. The results showed that higher levels of miR-155 and lower levels of IL-10 were found in intestinal B cells. Th2 cytokine IL-13 could increase the expression of miR-155 in B cells. Blocking miR-155 efficiently inhibited FA in mice, suggesting a therapeutic potential of miR-155 in the treatment of FA.

## RESULTS

### Intestinal B cells of FA mice show higher levels of miR-155

**Figure 1:**
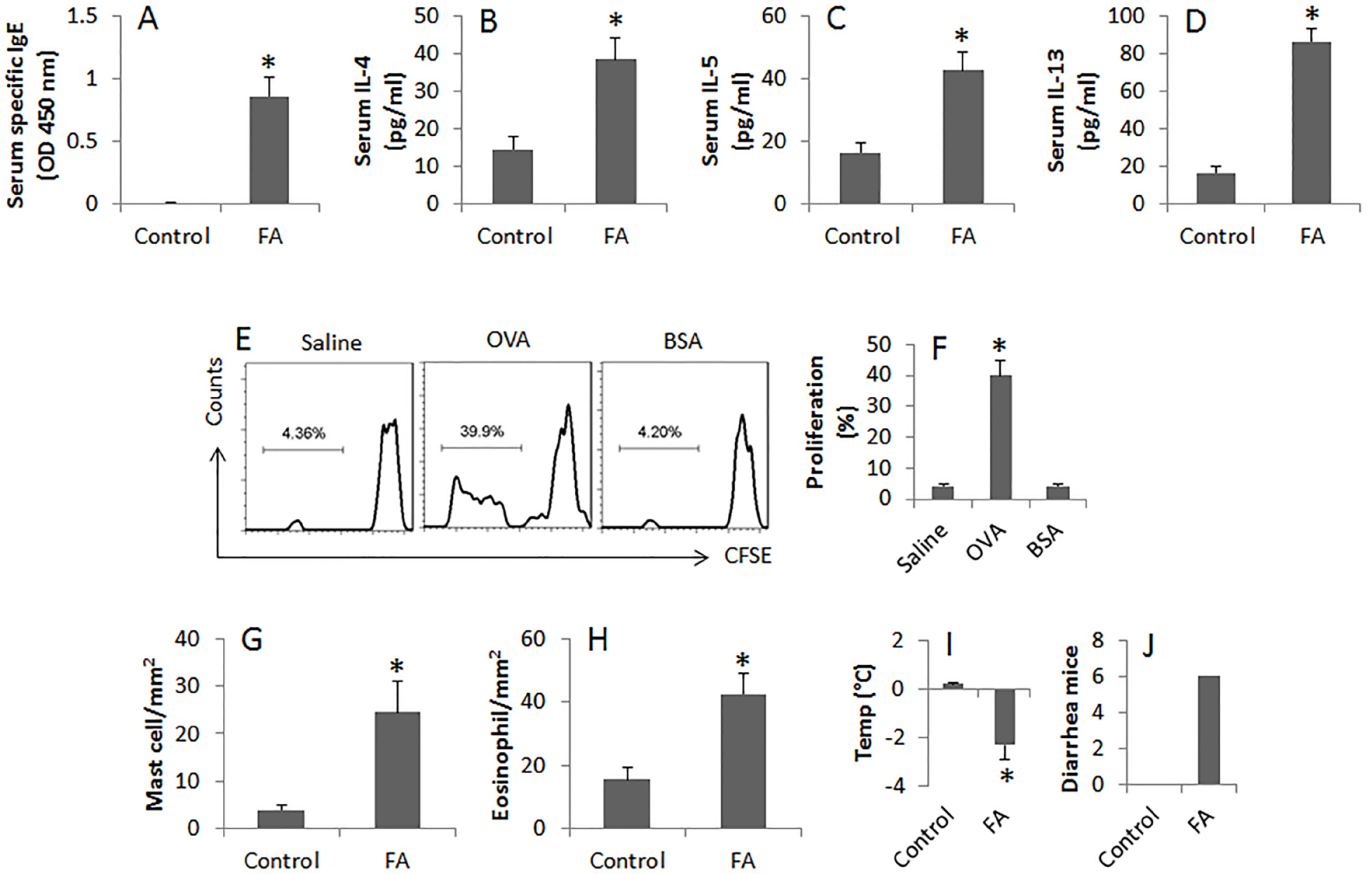
Assessment of allergen-related inflammation in the intestine **(A-D)**, the bars indicate the serum levels of specific IgE, IL-4, IL-5 and IL-13. The sera were collected from each mouse of control group and FA group at sacrifice. **(E)**, the flow cytometry histograms show the frequency of proliferating CD4^+^ T cells after exposure to saline or OVA or BSA in the culture for 72 h. **(F)**, the bars indicate the summarized data of E. **(G-H)**, the bars indicate the cell counts of mast cells and eosinophils in the intestinal tissue. **(I-J)**, the bars indicate the core temperature (Temp) changes and diarrhea mice 30 min after the last OVA challenge. Data of bars are presented as mean ± SD. *, p<0.01, compared with the control group. Each group consists of 6 mice. The data are representatives of 3 independent experiments.

**Figure 2:**
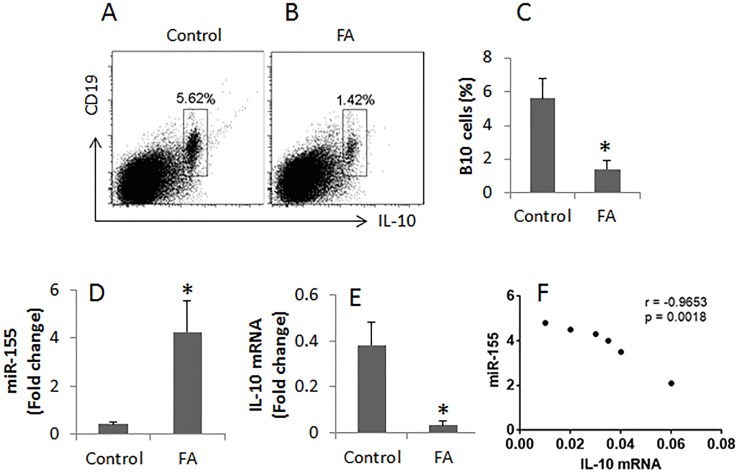
Assessment of IL-10^+^ B cells in the intestine **(A-B)**, the gated cell population shows the frequency of IL-10^+^ B cells in the lamina propria mononuclear cells from the control mice and the FA mice. **(C)**, the bars indicate the summarized data of A and B. **(D)**, the bars indicate the levels of miR-155 in B cells. **(E)**, the bars indicate the mRNA of IL-10 in intestinal B cells. **(F)**, the correlation between the expression of miR-155 and IL-10 in the intestinal B cells. Data of bars are presented as mean ± SD; *, p<0.01, compared with the control group. Each group consists of 6 mice. The data are representatives of 3 independent experiments.

To test the role of miR-155 in the pathogenesis of FA, we developed an FA mouse model. The mice showed high levels of specific IgE and Th2 cytokines in the sera, OVA-specific CD4^+^ T cells in the intestine, profound infiltration of mast cell and eosinophil in the intestinal mucosa. Upon the last challenge with OVA, mice showed a core temperature drop and diarrhea (Figure 1). The data indicate that the mice have FA-like inflammation in the intestine. In addition, we found less IL-10^+^ B cells (B10 cells) in the intestine (Figure 2A-2C); suggesting B10 cells were suppressed in the intestine.

To investigate the role of miR-155 in the regulation of B cell’s activities, we purified B cells from lamina propria mononuclear cells (LPMC). The RNA extracts were prepared from the B cells and analyzed by RT-PCR. The results showed significantly higher levels of miR-155 were detected in B cells from the FA mice as compared with the control mice (Figure 2D).

### FA mouse intestinal B cells express less IL-10 that is negatively correlated with miR-155

We next assessed the expression of IL-10 in intestinal B cells. The results showed markedly less IL-10 mRNA levels in intestinal B cells of FA mice as compared with control mice (Figure 2E). A correlation test was performed with the data of miR-155 and IL-10 of the intestinal B cells. The results showed a significant negative correlation (r = −0.9653, p<0.01) (Figure 2F) between miR-155 and IL-10 in the intestinal B cells.

### IL-13 up regulates miR-155 expression in B cells

**Figure 3:**
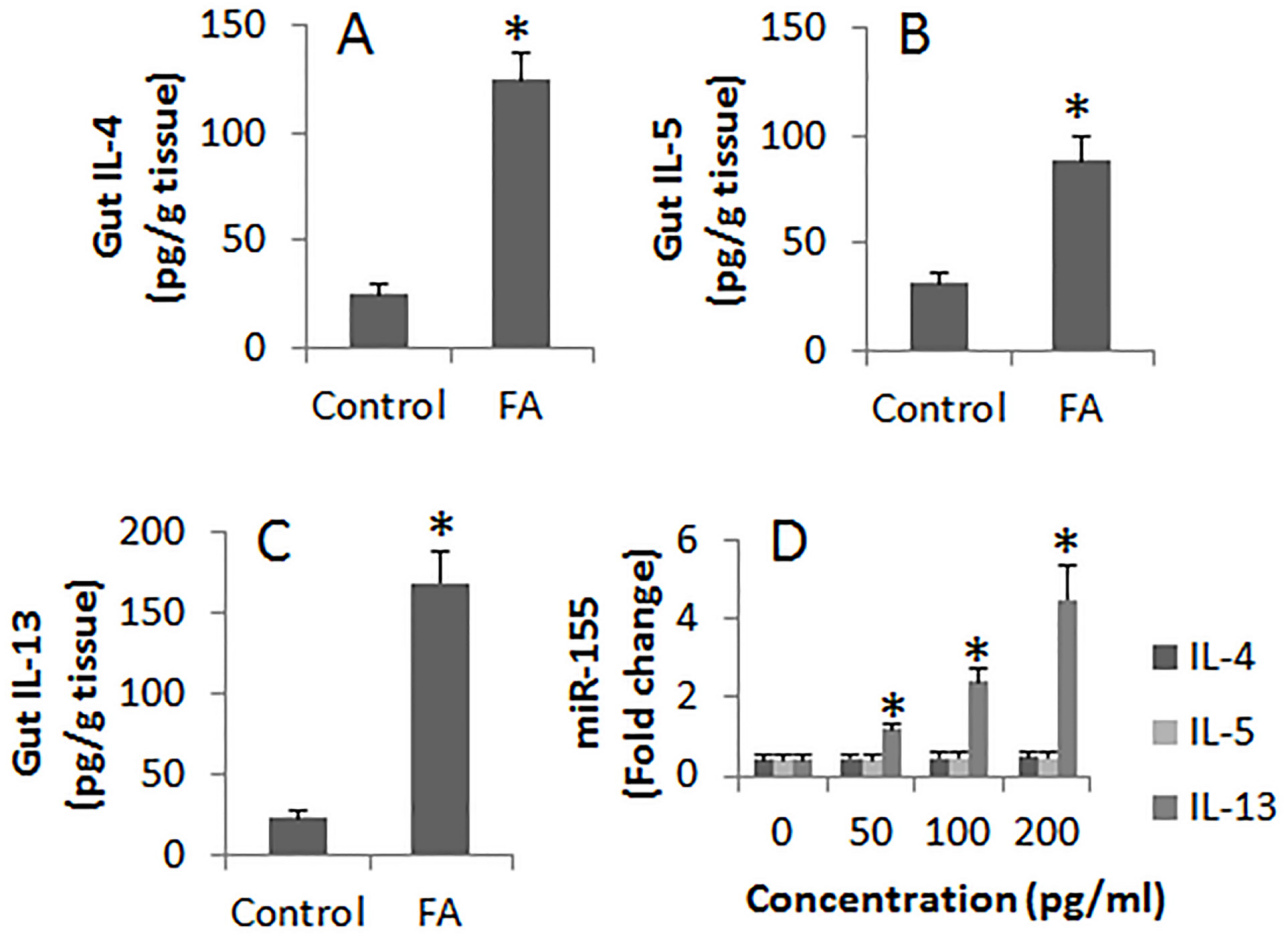
Assessment of Th2 cytokines in the intestine and their effects on the miR-155 expression in B cells **(A-C)**, the bars indicate the levels of Th2 cytokine in intestinal tissue extracts from control mice and FA mice. **(D)**, the bars indicate the miR-155 levels in B cells after stimulating by Th2 cytokines in the culture for 48 h. Data of bars are presented as mean ± SD; *, p<0.01, compared with the control group. The data are representatives of 3 independent experiments.

Considering the cytokines in the intestine may alter the expression of miR-155 in B cells of the intestinal mucosa, we firstly measured the Th2 cytokines in the intestinal protein extracts. The results showed that the levels of IL-4, IL-5 and IL-13 were significantly higher in FA mice than that in control mice. We then prepared B cells from the naive mouse spleen. The B cells were stimulated with IL-4 or IL-5 or IL-13 in the culture for 72 h. The B cells were harvested at the end of the culture and analyzed by RT-PCR. The expression of miR-155 was significantly up regulated in B cells after exposure to IL-13. Exposure to IL-4 or IL-5 also increased miR-155 slightly, but did not reach the significant levels (Figure 3).

### Blocking miR-155 attenuates IL-13-suppressed IL-10 expression in B cells and inhibits allergen-related inflammation in the intestine

**Figure 4:**
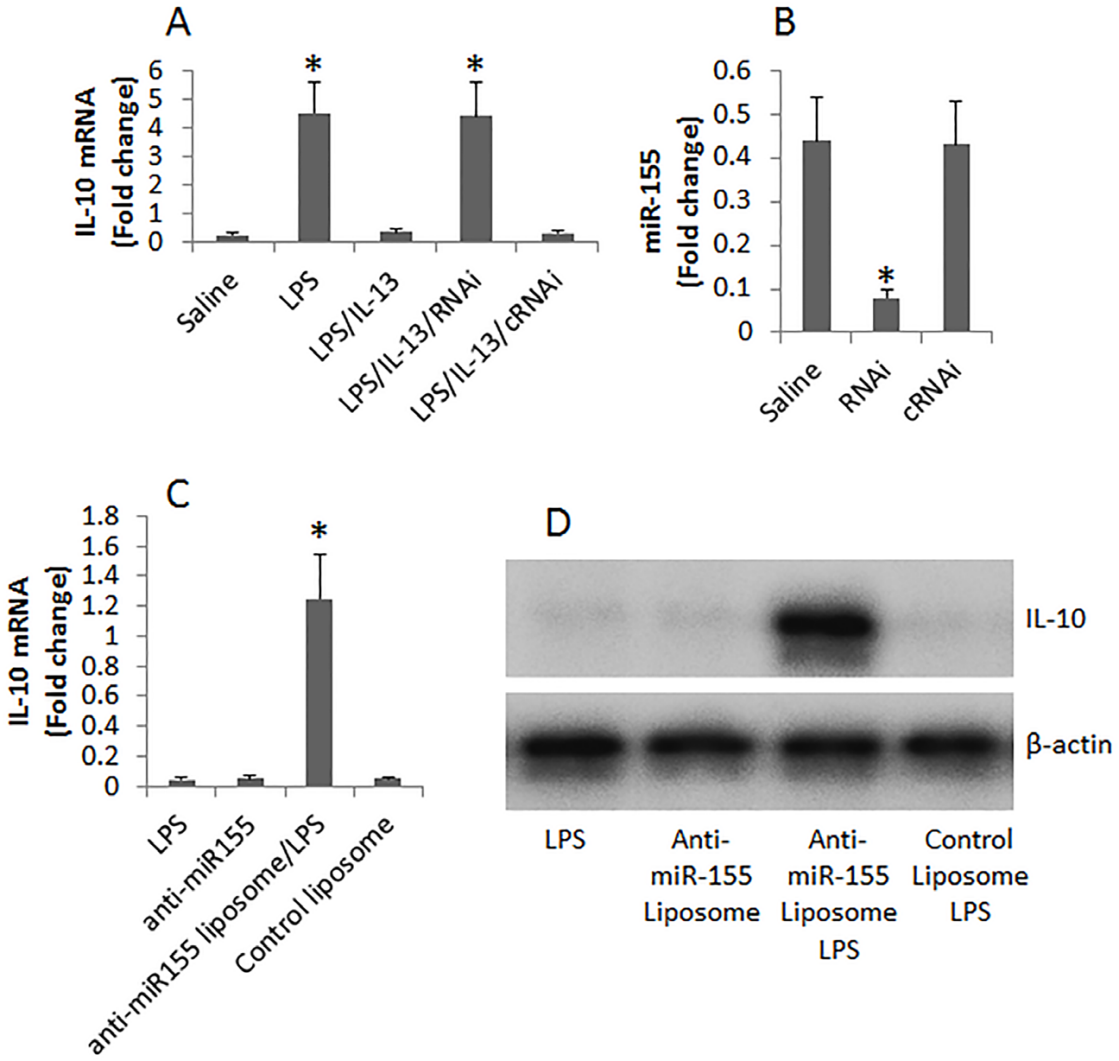
Assessment of the role of miR-155 in IL-13-suppressing IL-10 expression in B cells **(A)**, the bars show the levels of IL-10 mRNA in B cells after treating with the conditions denoted on the X axis. **(B)**, the bars show the levels of miR-155 in B cells after treating with saline or RNAi or control RNAi (cRNAi). RNAi: miR-155 RNAi. **(C-D)**, the expression of IL-10 in B cells after treating with anti-miR-155 or control in the presence of LPS. Data of bars are presented as mean ± SD; *, p<0.01, compared with the saline group. The data are representatives of 3 independent experiments.

Data reported above implicate that miR-155 may be involved in suppressing IL-10 expression in B cells. To test this, we employed an established cell culture model [14] to enhance the expression of IL-10 in B cells. We treated naive B cells with LPS in the presence or absence of IL-13 in the culture for 48 h. The B cells were then analyzed by RT-PCR. The results showed that exposure to LPS markedly enhanced the expression of IL-10 in B cells, which was abolished by the presence of IL-13 (Figure 4A). To test the role of miR-155 in the IL-13-suppressed IL-10 expression, we knocked down the miR-155 gene in B cells (Figure 4B). The miR-155-deficient B cells were exposed to lipopolysaccharide (LPS) and IL-13 in the culture for 48 h. Indeed, higher levels of IL-10 mRNA were detected in the B cells (Figure 4A). The data demonstrate that miR-155 is involved in the IL-13-suppressing IL-10 expression in B cells.

On the other hand, we isolated B cells from LPMC of FA mice. The cells were treated with LPS, or anti-miR-155 liposome and LPS, or control liposome and LPS for 2 days. The cells were then analyzed by RT-qPCR and Western blotting. The results showed that the presence of LPS alone or anti-miR-155 liposomes alone could not stimulate the B cells to produce IL-10. Exposure to both LPS and liposomes of anti-miR-155, but not the control liposomes, markedly increased the expression of IL-10 by the B cells. The results indicate that even if exposure to proper stimuli, B cells from the intestine of FA mice do not produce IL-10. Blocking miR-155 restores the ability of IL-10 production by the B cells.

**Figure 5:**
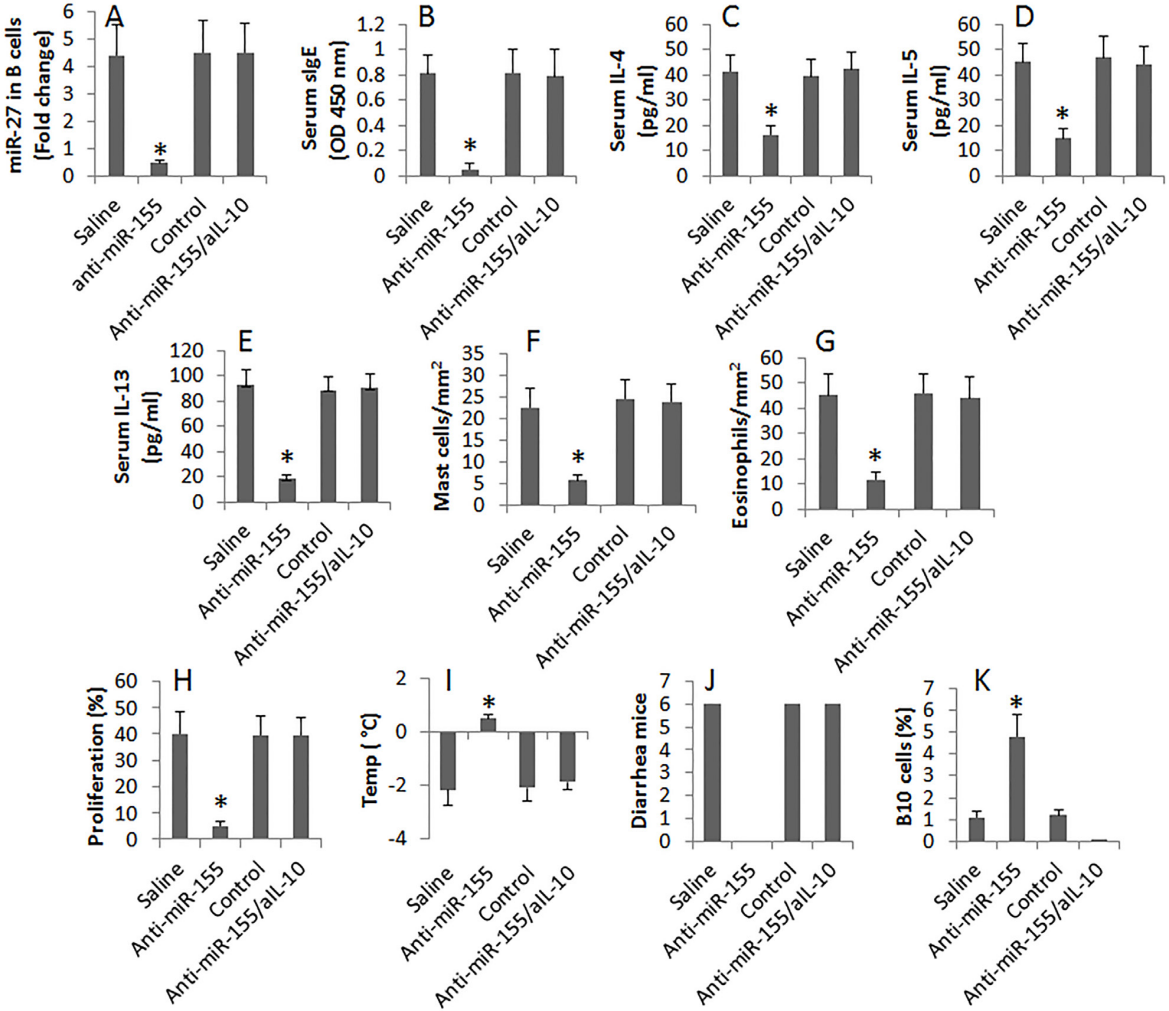
Anti-miR-155 therapy inhibits FA in mice FA mice were treated with the procedures listed on the X axis of each subpanel. Saline: Mice were treated with saline. Anti-miR-155: Mice were treated with the antisense of miR-155 liposomes. Control: Mice were treated with the empty liposomes. aIL-10: Mice were treated with anti-IL-10 antibody. **(A)**, the bars indicate the levels of miR-155 in intestinal B cells. **(B-K)**, the bars indicate the major FA parameters (B-J) and the frequency of B10 cells (K) in the intestinal mucosa of FA mice. Data of bars are presented as mean ± SD; *, p<0.01, compared with the saline group. Each group consists of 6 mice. Each experiment was repeated 3 times.

We next assessed the effects of inhibiting miR-155 on FA in mice. The mice were injected with the anti-miR-155-carrying liposomes for 3 times in the course of sensitization. The results showed that administration with anti-miR-155 liposomes efficiently suppressed FA in mice and increased the frequency of IL-10^+^ B cells in the intestine. To test if the anti-miR-155 treatment-recovered IL-10 production plays any roles in the anti-allergy effect, a group of FA mice were treated with anti-miR-155 and anti-IL-10 antibody. Indeed, the anti-allergy effect was abolished (Figure 5).

## DISCUSSION

This study has revealed a previously unknown phenomenon; miR-155 plays an important role in the development of intestinal allergy. We observed that in FA mice, the intestinal B cells showed higher levels of miR-155, which was correlated negatively with the IL-10 levels in the intestinal B cells. Th2 cytokines, including IL-4, IL-5 and IL-13, were higher in the intestinal mucosa; among which IL-13, but not IL-4 or IL-5, was capable of suppressing the expression of IL-10 in B cells that was dependent on up regulating miR-155 expression. Blocking miR-155 inhibited the IL-13-caused IL-10 suppression in B cells and inhibited the FA response in mice. The results suggest that inhibition of miR-15 may have the therapeutic capacity in the treatment of FA.

IL-10 is an important immune regulatory molecule. A number of studies indicate that IL-10-producing B cells are a critical cell population in the maintenance of the immune homeostasis in the body by suppressing abnormal immune responses. Yanabe et al reported that B10 cells inhibited contact hypersensitivity [15]. Amu et al indicated that B10 cells prevented and reversed allergic airway inflammation [16]. Mathsushita et al observed that B10 cells suppressed experimental autoimmune encephalomyelitis [17]. Roy et al found a decrease in IL-10 levels in splenocytes of FA mice [18]. Yanaba et al reported that IL-10-producing B10 cells inhibited intestinal inflammation in a mouse model [19]. The above information pin points that B10 cells are important in the inhibition of immune inflammation. The present data are in line with those pioneer studies by showing the lower frequency of B10 cells in the intestine of FA mice. Furthermore, from a correlation assay, we identified a negative correlation between the expression of IL-10 and miR-155 in intestinal B cells, implicating the high levels of miR-155 may be one of the causative factors of the suppression of IL-10 in B cells.

Billeter et al found that miR-155 negatively regulates IL-10 production in macrophages by targeting the 3'untranslated region of the IL-10 transcript [13]. We also found that miR-155 inhibited the LPS-increased IL-10 expression in B cells. On the other hand, we further identified a causative factor for increasing miR-155 that exposure to IL-13 increased the expression of miR-155 and then suppressed IL-10 expression in B cells. Our previous studies also found that IL-13 suppressed the expression of thrombospondin-1 (TSP1) in B cells to compromise the immune tolerance in the intestine [20]. Whether the inhibition of TSP1 by IL-13 is also mediated by miR-155 needs further investigation.

In summary, the data demonstrate that miR-155 plays a role in the initiation of FA in mice. When the miR-155 gene was knocked down in B cells, the IL-13-suppressed IL-10 expression in B cells was abolished. Using a miR-155 inhibitor could inhibit FA response in mice. Thus, we conclude that miR-155 is a critical factor in the initiation of FA in mice. Blocking miR-155 has the therapeutic potential for the treatment of FA.

## MATERIALS AND METHODS

### Reagents

The ELISA kit of OVA-specific IgE was purchased from AbD Serotec (Raleigh, NC); ELISA kits and proteins of IL-4, IL-5 and IL-13 were purchased from R&D Systems (Minneapolis, MN). The fluorochrome-labeled antibodies of CD19 (SJ25C1) and IL-10 (JES319F1) for flow cytometry were purchased from BD Biosciences (Franklin Lakes, NJ). The anti-IL-10 polyclonal antibody (V-15) was purchased from Santa Cruz Biotech (Santa Cruz, CA). The reagents for RT-qPCR and Western blotting were purchased from Invitrogen (Carlsbad, CA). The reagents for constructing the liposomes were purchased from Sigma Aldrich (St. Louis., MO). The reagent kits for immune cell isolation were purchased from Miltenyi Biotech (San Diego, CA).

### Mice

Male BALB/c mice (6-8 week old) were purchased from the Xinmao Experimental Animal Center (Shanghai, China). Mice were maintained in a pathogen-free environment under a 12:12 h light-dark cycle. The mice were allowed to access food and water freely. The experimental procedures were approved by the Animal Ethic Committee at Tongji University. The experiments were performed in accordance with the approved procedures and guidelines.

### Development of FA mouse model

Following our established procedures [21], grouped mice (6 per group) were gavage-fed with ovalbumin (OVA, 1 mg/mouse) and cholera toxin (20 μg/mouse) mixed in 0.3 ml saline once a week for 4 consecutive weeks. The mice were challenged with OVA (5 mg/mouse) by gavage-feeding on the fifth week and sacrificed 60 min after the challenge.

### Assessment of FA response

The core temperature was recorded from the anus of mouse 30 min after OVA challenge. Diarrhea was recorded within 1 h after the challenge. The blood and the small intestine were collected immediately after sacrifice.

#### Assessment of Th2 cytokines in the sera and intestinal tissue

The sera were isolated from the collected blood samples by centrifugation. The proteins were extracted from a segment of the jejunum. Th2 cytokines, including IL-4, IL-5 and IL-13, and specific IgE in the sera and intestinal tissue were determined by enzyme-linked immunosorbent assay (ELISA) with purchased reagent kits (R&D Systems and Biomart, Shanghai, China) following the manufacturer’s instructions.

#### Detection of antigen-specific CD4^+^ T cells in the intestine

The small intestine was excised from mice after sacrifice. Lamina propria mononuclear cells (LPMC) were isolated from the intestinal tissue following our established procedures [22]. CD4+ T cells and dendritic cells (DC) were purified from the LPMCs by magnetic cell sorting (MACS) with commercial reagent kits (Miltenyi Biotech) following the manufacturer’s instructions. The CD4^+^ T cells (labeled with CFSE; carboxyfluorescein diacetatesuccinimidyl ester) and DCs were cocultured at a ratio of 10^5^ CD4^+^ T cells:2 × 10^4^ DCs/well in the presence of specific antigen OVA (5 μg/ml) or bovine serum albumin (BSA, 5 μg/ml; used as an irrelevant antigen) for 3 days. The cells were analyzed by flow cytometry. The proliferating cells were regarded as the antigen-specific CD4^+^ T cells.

#### Counts of mast cell/eosinophil in the intestinal tissue

A segment of the jejunum was fixed with 4% formalin overnight and processed for paraffin sections. The sections were stained with 0.5% toluidine blue (for mast cell staining) or hematoxylin/eosin (for eosinophil staining). Mast cells and eosinophils on the sections were counted under a light microscope. Randomly selected 20 fields (× 400) were counted for each sample. The sections were coded. The observers were not aware of the code to avoid the observer bias.

### Assessment of the frequency of B10 cells in the intestinal tissue by flow cytometry

The isolated LPMCs were stained with anti-CD19 antibody (FITC labeled; BD Bioscience) for 30 min at 4°C. After washing with phosphate buffered saline (PBS) for 3 times, the cells were fixed with 1% paraformaldehyde for 30 min and 0.5% saponin for another 30 min. The cells were incubated with anti-IL-10 antibody (PE labeled; BD Bioscience) for 30 min at 4°C. After washing, the cells were analyzed with a flow cytometer (FACSCanto II; BD Bioscience). The CD19^+^ IL-10^+^ cells were regarded as B10 cells. Isotype IgG was used as a negative control antibody. Data of isotype IgG staining were used as a gating reference.

### Isolation of B cells and cell culture

B cells were isolated from the prepared LPMC with purchased reagent kits following the manufacturer’s instructions. The purity of isolated B cells was 92%-96% as checked by flow cytometry. The B cells were cultured in RPMI1640 medium supplemented with 10% fetal bovine serum, 100 U/ml penicillin, 0.1 mg/ml streptomycin, 2 mM L-glutamine and 20 ng/ml anti-CD40 antibody (to avoid B cell apoptosis). The medium was changed in 2-3 days. The cell viability was greater than 99% as assessed by the Trypan blue exclusion assay.

### Assessment of miR-155 and IL-10 mRNA in B cells

CD19^+^ B cells were isolated from LPMCs or spleen cells by MACS with commercial reagent kits (Miltenyi Biotech) following the manufacturer’s instructions. The total RNAs were extracted from the B cells with TRIzol reagents (Invitogen). The cDNA was synthesized with the RNA and the PrimeScript™ RT reagent Kit (Invitrogen). The samples were analyzed by real time PCR using SYBR Green Master Mix (Invitrogen). Reference gene RNA U6B (Invitrogen) was used as an internal control. The results were calculated with the 2^−Δ ΔCt^ method and presented as fold change against controls. The primers of miR-155 were provided by Qiagen. The primers of IL-10 are ggtgagaagctgaagaccct and tgtctaggtcctggagtcca. The primers of β-actin are gtgggaatgggtcagaagga and tcatcttttcacggttggcc.

### Assessment of the effects of Th2 cytokine on the expression of miR-155 in B cells

B cells were isolated from naïve mouse spleen by MACS as described above. The B cells were cultured in microplates (10^5^ cells/well) in RPMI1640 medium supplemented with 10% fetal bovine serum, 100 U/ml penicillin, 0.1 mg/ml streptomycin and 2 mM L-glutamine. IL-4 or IL-5 or IL-13 was added to the culture at gradient concentrations (0-200 pg/ml) for 48 h. The B cells were harvested at the end of culture and subjected to an assessment of miR-155 expression by real time RT-PCR as described above. Each sample was analyzed in triplicate.

### Up regulation of IL-10 expression in B cells

B cells were isolated from naïve mouse spleen by MACS as described above. Lipopolysaccharide (LPS; 1 μg/ml) was added to the culture for 48 h. The levels of IL-10 mRNA in the B cells were analyzed by real time RT-PCR as described above.

### Assessment of the effects of IL-13 on IL-10 expression in B cells

As described above, B cells were stimulated by LPS to up regulate the expression of IL-10. In separate experiments, IL-13 (200 pg/ml) was added to the culture together with LPS for 48 h. The expression of IL-10 by the B cells was analyzed by real time RT-PCR.

### RNA interference (RNAi) of miR-155

The RNAi reagent kit of miR-155 was provided by Beijing Yijie Biotech (Beijing, China). The miR-155 RNAi was performed with B cells with the reagent kit following the manufacturer’s instructions. The RNAi effect on the B cells was assessed by RT-qPCR.

### Preparation of anti-miR-155 liposome

Following published procedures [23], 13 μmol of a lipid mixture (Sigma Aldrich) in 200 μL of 100% ethanol was slowly added under strong vortex to 0.086 μmol of anti-miR-155 oligonucleotides (Beijing Yijie Biotech; Beijing, China) in 300 μL of 20 mM citrate buffer (pH 4) (the solutions were heated to 60°C). The resulting particles were extruded 13 times through a polycarbonate membrane (100-nm-diameter) using a LiposoFast basic extruder (Avestin, Toronto, Canada). A Sepharose CL-4B column was prepared and equilibrated with HBS pH 7.4. The samples were passed through the column to remove ethanol and nonencapsulated anti-miR-155. The total lipid concentration was assessed by cholesterol quantification using the Liebermann–Burchard test [24].

### Inhibition of miR-155 in mice with anti-miR-155 liposomes

To test the effects of the anti-miR-155 liposome on suppression of miR-155 in B cells, in the course of sensitization, mice were i.p. injection with anti-miR-155 liposome (0.1 mg/mouse) one day prior to each exposure to OVA and CT. The procedures of miR-155 and FA response assessment are the same as described above.

### Blocking the effects of IL-10 in mice

FA mice were injected with anti-IL-10 polyclonal antibodies at 0.25 mg/mouse one day prior to each exposure to OVA and CT.

### Statistics

Data are presented as mean ± SD. The difference between two groups was tested by Student t test or ANOVA if more than two groups. P<0.05 was regarded as significant.

## References

[1] Devereux G (2006). The increase in the prevalence of asthma and allergy: food for thought. Nat Rev Immunol.

[2] Elsenbruch S, Enck P (2015). Placebo effects and their determinants in gastrointestinal disorders. Nat Rev Gastroenterol Hepatol.

[3] Deschildre A, Elegbede CF, Just J, Bruyere O, Van der Brempt X, Papadopoulos A, Beaudouin E, Renaudin JM, Crepet A, Moneret-Vautrin DA (2016). Peanut-allergic patients in the MIRABEL survey: characteristics, allergists' dietary advice and lessons from real life. Clin Exp Allergy.

[4] Imanzadeh F, Nasri P, Sadeghi S, Sayyari A, Dara N, Abdollah K, Nilipoor Y, Mansuri M, Khatami K, Rouhani P, Olang B (2015). Food allergy among Iranian children with inflammatory bowel disease: a preliminary report. J Res Med Sci.

[5] Kloster Smerud H, Fellstrom B, Hallgren R, Osagie S, Venge P, Kristjansson G (2010). Gastrointestinal sensitivity to soy and milk proteins in patients with IgA nephropathy. Clin Nephrol.

[6] Chinthrajah RS, Hernandez JD, Boyd SD, Galli SJ, Nadeau KC (2016). Molecular and cellular mechanisms of food allergy and food tolerance. J Allergy Clin Immunol.

[7] Tanoue T, Atarashi K, Honda K (2016). Development and maintenance of intestinal regulatory T cells. Nat Rev Immunol.

[8] Lund FE, Randall TD (2010). Effector and regulatory B cells: modulators of CD4+ T cell immunity. Nat Rev Immunol.

[9] Liu ZQ, Wu Y, Song JP, Liu X, Liu Z, Zheng PY, Yang PC (2013). Tolerogenic CX3CR1+ B cells suppress food allergy-induced intestinal inflammation in mice. Allergy.

[10] Lee SJ, Noh G, Lee JH (2013). In vitro induction of allergen-specific interleukin-10-producing regulatory B cell responses by interferon-gamma in non-immunoglobulin e-mediated milk allergy. Allergy Asthma Immunol Res.

[11] Noh J, Lee JH, Noh G, Bang SY, Kim HS, Choi WS, Cho S, Lee SS (2010). Characterisation of allergen-specific responses of IL-10-producing regulatory B cells (Br1) in Cow Milk Allergy. Cell Immunol.

[12] Cortez MA, Bueso-Ramos C, Ferdin J, Lopez-Berestein G, Sood AK, Calin GA (2011). MicroRNAs in body fluids—the mix of hormones and biomarkers. Nat Rev Clin Oncol.

[13] Billeter AT, Hellmann J, Roberts H, Druen D, Gardner SA, Sarojini H, Galandiuk S, Chien S, Bhatnagar A, Spite M, Polk HC (2014). MicroRNA-155 potentiates the inflammatory response in hypothermia by suppressing IL-10 production. Faseb j.

[14] Villagra A, Cheng F, Wang HW, Suarez I, Glozak M, Maurin M, Nguyen D, Wright KL, Atadja PW, Bhalla K, Pinilla-Ibarz J, Seto E, Sotomayor EM (2009). The histone deacetylase HDAC11 regulates the expression of interleukin 10 and immune tolerance. Nat Immunol.

[15] Yanaba K, Bouaziz JD, Haas KM, Poe JC, Fujimoto M, Tedder TF (2008). A regulatory B cell subset with a unique CD1dhiCD5+ phenotype controls T cell-dependent inflammatory responses. Immunity.

[16] Amu S, Saunders SP, Kronenberg M, Mangan NE, Atzberger A, Fallon PG (2010). Regulatory B cells prevent and reverse allergic airway inflammation via FoxP3-positive T regulatory cells in a murine model. J Allergy Clin Immunol.

[17] Matsushita T, Yanaba K, Bouaziz JD, Fujimoto M, Tedder TF (2008). Regulatory B cells inhibit EAE initiation in mice while other B cells promote disease progression. J Clin Invest.

[18] Roy R, Kumar S, Verma AK, Sharma A, Chaudhari BP, Tripathi A, Das M, Dwivedi PD (2014). Zinc oxide nanoparticles provide an adjuvant effect to ovalbumin via a Th2 response in Balb/c mice. Int Immunol.

[19] Yanaba K, Yoshizaki A, Asano Y, Kadono T, Tedder TF, Sato S (2011). IL-10-producing regulatory B10 cells inhibit intestinal injury in a mouse model. Am J Pathol.

[20] Yang G, Geng XR, Liu ZQ, Liu JQ, Liu XY, Xu LZ, Zhang HP, Sun YX, Liu ZG, Yang PC (2015). Thrombospondin-1 (TSP1)-producing B cells restore antigen (Ag)-specific immune tolerance in an allergic environment. J Biol Chem.

[21] Yang B, Li LJ, Xu LZ, Liu JQ, Zhang HP, Geng XR, Liu ZG, Yang PC (2016). Histone acetyltransferease p300 modulates TIM4 expression in dendritic cells. Sci Rep.

[22] Yang PC, Xing Z, Berin CM, Soderholm JD, Feng BS, Wu L, Yeh C (2007). TIM-4 expressed by mucosal dendritic cells plays a critical role in food antigen-specific Th2 differentiation and intestinal allergy. Gastroenterology.

[23] Costa PM, Cardoso AL, Custódia C, Cunha P, Pereira de Almeida L, Pedroso de Lima MC (2015). MiRNA-21 silencing mediated by tumor-targeted nanoparticles combined with sunitinib: A new multimodal gene therapy approach for glioblastoma. J Control Release.

[24] Costa PM, Cardoso AL, Mendonca LS, Serani A, Custodia C, Conceicao M, Simoes S, Moreira JN, Pereira de Almeida L, Pedroso de Lima MC (2013). Tumor-targeted chlorotoxin-coupled nanoparticles for nucleic acid delivery to glioblastoma cells: a promising system for glioblastoma treatment. Mol Ther Nucleic Acids.

